# Notes from Dental Practice

**Published:** 1869-05

**Authors:** 


### ARTICLE VI.

### *Notes from Dental Practice.*

CASE. 1.—*Treatment of Exposed Pulps.* Nature of case. —The cavity of decay on the anterior approximal surface of a superior second bicuspid tooth, the removal of the decomposed dentine exposing the pulp which was found to be in a perfectly healthy condition.

Treatment.—After carefully removing the carious portion and giving a proper form to the cavity for the retention of the filling, the next step inthe operation was the protection of the pulp. For this purpose recourse was had to the oxychloride of zinc, which was prepared by combining the powdered oxide with the liquid chloride in the form of a thick paste.

These preparations of zinc should be of the best quality, and thoroughly mixed together, so as to form a paste which does not present a watery appearance upon the surface; care must also be observed that the paste does not commence to solidify before it is introduced

In order that no time might be lost after the mixing of this paste to the proper consistency (as it rapidly hardens), the cavity was first dried, and then carefully protected from moisture by requesting the patient to keep the napkin in place about it with his fingers. The paste as soon as prepared was applied directly over the exposed pulp on a small piece of soft linen of a size corresponding to the bottom of the cavity, both surfaces of this piece of linen being coated with it.

After the introduction of the piece of linen, the cavity over it was completely filled with the paste, and this temporary filling protected from moisture for about twenty minutes, this time being necessary for the proper hardening of the material. The surface of the filling was then made smooth with a burnisher, and to protect it for a still longer time from moisture, was painted over with a coating of sandarach varnish. Collodion also answers a good purpose for thus protecting the surface; these directions applying more especially to temporary fillings of these preparations of zinc, which are intended to remain in the teeth for some months.

An engagement was then made with the patient for the following week at which time it was determined to permanently fill the tooth should no untoward symptoms arise.

The tooth remaining perfectly quiet from the time the temporary filling was introduced, until that of the second engagement, the method pursued was as follows: All of the temporary filling, composed of the oxy-chloride of zinc, was removed, except that portion of it covering the bottom of the cavity, and immediately over the pulp, care being taken not to cut through this or in any way to injure it. When this was accomplished a gold filling was introduced by hand-pressure (as it was deemed unadvisable to use mallet-force in this instance), and the cavity thus permanently secured.

The application of the paste to the exposed surface of the pulp at the time of the introduction of the temporary filling, was followed by some pain, which, however, soon subsided.

This treatment of an exposed pulp, only promises success in cases where the organ is in a perfectly healthy condition, free from inflammation, or injury occurring in removing the decay. Where the exposed pulp is in a state of irritation palliative treatment should first be resorted to, and that above described be pursued when the former has proved successful.

Case 2. *A Peculiar Form of Ulceration of the Gums.*

Nature of case.—Patient a youth aged 19 years who from childhood has been afflicted with diseased gums, the symptoms of the affection being as follows: The gums have a pale red and white color, are not painful but constantly discharging a fetid matter from about the necks of the teeth. They are not well festooned, and become irritable from slight causes; the patient also complains that in an hour after cleansing his teeth with the brush, they (the teeth) become dark and unsightly. This peculiar form of gingivitis was first described by Professor Harris, and has a constitutional origin, requiring constitutional as well as local treatment.

Treatment.—Constitutional.—One-half drachm of Chlorate of Potash was administered daily in divided doses of ten grains each, largely diluted as this salt is not very soluble.

Local.—During the administration of the Chlorate of Potash, the following local treatment was pursued: The edges of gums, about the necks of the teeth, were touched with a solution of Nitrate of Silver, three grains to the ounce of water, and the following gargle used frequently during the day: ℞. Potass. chlor. ʒ ij. Tinct. Catechu f. ʒ ij. Cologne water f. ℥ j. Aqua ℥ vj. Misce. This treatment was successful in arresting the progress of the affection, and so decided an improvement in the condition of the mouth has taken place that the patient, having discontinued the use of the Chlorate of Potash gargle, is using a simple astringent one composed of Borax ʒ j. Tinct. Myrrh f. ℥ ss. Honey f. ℥ j. Rose Water f. ℥ iv. Misce.

